# Private psychiatric care in the past: With special reference to Chennai

**DOI:** 10.4103/0019-5545.39765

**Published:** 2008

**Authors:** O. Somasundaram

**Affiliations:** Thanigai Illam, Besant Nagar, Chennai, Tamil Nadu, India

## Abstract

The ‘madhouse’ managed by Connolly and Dalton, situated in Chennai, prior to the opening of the Madras Lunatic Asylum in 1871 is described. The status of the private madhouses of England that existed before the county asylums were established in 1845 is briefly touched upon. A legitimate criticism of the shortcomings of this system along with the need for reorganization was forcibly brought by writers such as Defoe and others. Their suggestions find a place in subsequent mental health legislations. The legislation covering these aspects incorporated in the Mental Health Act 1987 form the basis for the licensing of our private mental health care centres.

## THE PRIVATE SECTOR IN THE PSYCHIATRIC CARE (WITH SPECIAL REFERENCE TO TAMIL NADU)

In ancient times and the Middle Ages, most of the ‘harmless’ mentally ill were cared for in the families themselves or allowed to fend for themselves in the community. Various religious orders were sympathetic and allowed them boarding and lodging. Separate hospitals for them were almost unknown, with a few remarkable exceptions: Bedlam in London, Almansur in Cairo, Thirumukkudal in Chengalpattu district, Tamil Nadu.[1]

The care of the violent and aggressive patients posed difficult problems. Some ‘lay specialists’ accommodated them under their care with a few attendants to look after them, with or without restraints. Some unwanted family members who were troublesome in any way or whose properties could be coveted in their absence; drunkards or the sexually promiscuous, etc. were conveniently moved to the so-called ‘private madhouses’. These houses played a remarkable role in psychiatric care and many of the proprietors of these houses were doing a lucrative ‘trade in lunacy’ before lunatic asylums were established under state control, such as the county asylums of the UK in the 19^th^ century.

The Madras Lunatic Asylum was sanctioned by the then government at the erstwhile Madras Presidency in G.O. No. 26 Judicial, dated 7^th^ January 1867. The asylum was constructed on a 66½-acre site in Locock's Garden outside the municipal limits. It started functioning on 15^th^ May 1871 with 145 patients and Surgeon John Murray MD as Superintendent with residential quarters inside the premises.[2] Before this arrangement for psychiatric care in a government institution, the mentally ill of European origin and Eurasians(Anglo-Indians) were cared for in a private madhouse.

The leading characters in this area were two British medical officers, Valentine Connolly and J. Dalton. (It is not proposed to deal here with two other British Presidencies, Bengal and Bombay. There are remarkable and complex differences in these Administrations and the reader is referred to articles by Ernst.)[3,4]

The roots of the hospital were planted in 1795 when the East India Company was the Authority. It appointed Surgeon Valentine Connolly in charge. Premises were taken on lease for a period of 20 years at a monthly rent of Rs. 825. Subsequently, Surgeon Maurice Fitzgerald held charge till 1803. Dr. Dalton then rebuilt it and it was called ‘Dalton's Mad Hospital’. When he retired, 54 inmates were being taken care of in it.

That the motives of the madhouse proprietors of Madras were not all altruistic, humane and well-meaning could be surmised from Ernst's views:

“The accounts praising Connolly's achievements are divided between mention of personal profit on one hand and public benevolence on the other. On his return to England he had accumulated great wealth and was acknowledged as one of those formerly less-well-off Englishmen who returned from India as wealthy nawab. He settled down in London, comfortably seeing his five sons through education in prestigious colleges and thus preparing them for promising careers as military officers and members of the Civil Service in India. Before embarkation, Connolly had sold the asylum buildings for three times the premises’ estimated value to another medical practitioner who expected the asylum to be a good enough income source to enable him to imitate his predecessor's rise to fortune.”

The achievements of Dr. Dalton do not appear to be praiseworthy or laudatory either. To quote Ernst once again:

“Until 1815 government did not get involved in lunacy trade. In 1815 when Surgeon J. Dalton considered selling the place for a highly inflated price, it was held that ‘the principle of selling not merely the building but the charge of the patients contained in it, to any individual however qualified or personally unobjectionable’ could be sanctioned by government (Madras Military Despatch 1823). Surgeon Dalton was consequently not only stuck with the asylum but on his retirement to Europe had also to hand over the medical charge of the institution to a surgeon who was henceforth appointed by government on recommendation from the Medical Board.”

It could be seen that proprietors in charge of the madhouses of Madras City of the 18^th^ and 19^th^ centuries were not very different from their counterparts of the same period in the UK. It may not be out of place to mention a few details about these madhouses of the same period in the UK.

It would be interesting to know that much care has been taken to regulate the private sector of psychiatric services. The history of these “shady but indispensable service providers”[5] is worth recapitulating at this juncture. Before 1774, there were no legal provisions for their control. Daniel Defoe's demand was:[6]

“In my humble opinion, all private Mad-Houses should be suppressed at once and it should be no less than felony to confine any person under pretence of Madness without due authority. For the care of those who are really Lunatick, licens'd Mad-Houses should be constituted in convenient parts of the Town, which Houses should be subject to proper visitation and inspection; nor should any person be sent to a Mad-House without due Reason, Inquiry and Authority.”

Another forcible statement along these lines was made by a pamphlet of 1740:

“Several are put into Mad-Houses as they are called without being mad. Wives put their Husbands in them that they may enjoy their Gallants and live without the Observation and Interruption of their Husbands; Husbands put their Wives in them, that they may enjoy their Whores without disturbance from their Wives; Children put their Parents in them that they may enjoy their Estates before their time; Relations put their Kindred into them for wicked purposes.”

It had also not escaped the notice of the shrewd observers of the madhouses that their proprietors were interested in prolonging the stay of their patients. Faulkner advocated splitting the ‘hotel’ function from the clinical:[7]

“The madhouse keeper should solely provide accommodation. The patient and his family should have free choice of practitioner; let them consult a Fordyce, a Baker, a Warren or a Reynolds, men whose knowledge cannot be surpassed, whose integrity is unimpeachable and who can derive no advantage from the local situation of the afflicted.”

Legal control and the medical supervision of the madhouses were introduced subsequently.[8] The Act of 1774 made provisions for the licensing and inspection of private madhouses within a radius of 7 miles thereof, by five Commissioners appointed by the College of Physicians and accompanied by a medical practitioner. The Mad-House Act of 1828 made improper detention of lunatics difficult. It could be seen that the regulations of madhouses of 18^th^ - and 19^th^ -century England are the beacon lights for the regulation of our Mental Nursing Homes of the present day.

To reduce the malpractice almost natural to the madhouse system, the Mental Health Act of India 1987 has taken enormous care with their regulation. They are dealt with in Chapters III and IV of the Act. The powers are delegated to the various states in the State Mental Health Rules.

**Figure 1:**
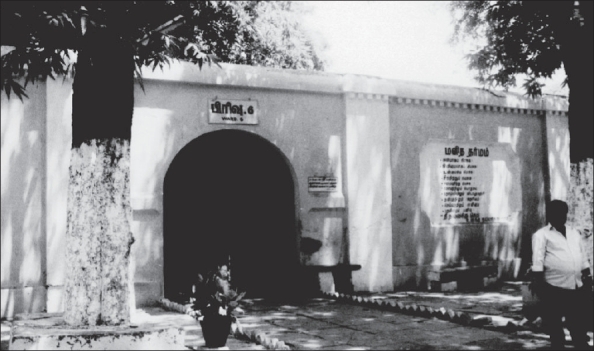
Security Block for Criminal Patients (Built 1871)

**Figure 2:**
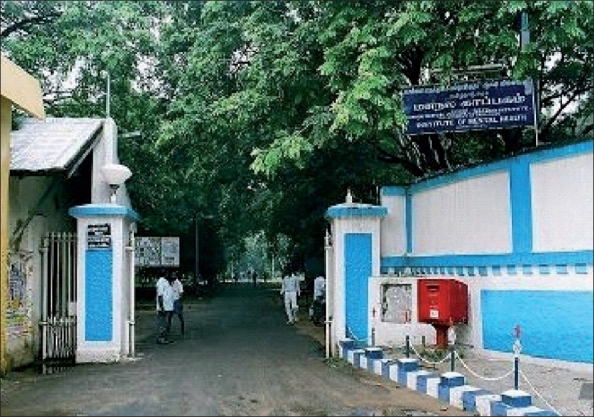
Entrance: Government Mental Hospital, Kilpauk, Chennai (Built 1871)

**Figure 3:**
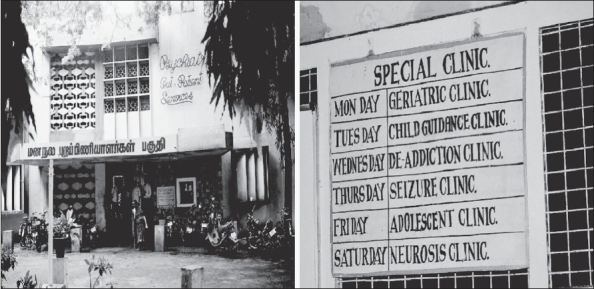
New Outpatients’ Block (Built 1971)

## References

[1] Somasundaram O (1987). Presidential address: The Indian Lunacy Act 1912: The Historic Background. Indian J Psychiatry.

[2] Subramaniam N, Somasundaram O The Government Mental Hospital, Madras - A Short History 1971. Memoirs of the Fifties.

[3] Ernst W, Bynum WP, Porter R, Shepherd M (1998). Asylum in alien places: The treatment of the European insane in British India. The Anatomy of Madness.

[4] Ernst W, Berrios GE, Hugh-Freeman (1991). Colonial psychiatry: The European Insane in British India 1800-58. 150 Years of British Psychiatry 1841-1991.

[5] Porter R (1990). Mind-forg'd Manacles.

[6] Defoe D (1697). An Essay upon Projects.

[7] Faulkner B (1789). Observation on the General and Improper Treatment of Insanity.

[8] Parry-Jones WL (1972). The Trade in Lunacy. Routledge and Keegan-Paul.

[9] Nambi S (1996). Legal aspects of psychiatry. Indian J Psychol Med.

